# Exercise increases serum endostatin levels in female and male patients with diabetes and controls

**DOI:** 10.1186/1475-2840-13-6

**Published:** 2014-01-06

**Authors:** Michael Sponder, Daniela Dangl, Stephanie Kampf, Monika Fritzer-Szekeres, Jeanette Strametz-Juranek

**Affiliations:** 1 Department of Cardiology, Institute of Internal Medicine II, Medical University of Vienna, Währinger Gürtel 18-20, Vienna 1090, Austria; 2 Department of Anaesthesiology, Intensive Care and Pain Management, Medical University of Vienna, Währinger Gürtel 18-20, Vienna 1090, Austria; 3 Department of Medical-Chemical Laboratory Analysis, Medical University of Vienna, Währinger Gürtel 18-20, Vienna 1090, Austria; 4 Department of Cardiology, Medical University of Vienna, Währinger Gürtel 18-20, Vienna 1090, Austria

**Keywords:** Diabetes, Exercise, Endostatin, Atherosclerosis, Sex

## Abstract

**Background:**

Type 2 diabetes mellitus (T2DM) is often associated with atherosclerotic changes in coronary vessels, most notably plaques. The angiostatic parameter endostatin is able to inhibit angiogenesis in tissue as well as in plaques and therefore plays an important role in physiological and pathological neovascularisation. The aim of the present study was to investigate sex-specific differences and the influence of exercise on circulating endostatin levels in patients suffering from diabetes, and control subjects.

**Methods:**

In total, 42 T2DM-patients and 45 control subjects were investigated. They underwent a graded physical stress test (ergometry). Serum endostatin levels were measured in venous blood at rest and directly after reaching maximum workload.

**Results:**

Females showed significantly higher endostatin levels at baseline measurements compared to men, independently of their underlying disease. In both female and male T2DM-patients endostatin levels were significantly lower compared to controls. Both groups and sexes showed a significant increase of endostatin after physical stress, whereas the extent of endostatin-increase was between 10.59-15.05%.

**Conclusion:**

Middle-aged healthy female individuals as well as female T2DM-patients showed higher circulating serum endostatin levels compared to males, suggesting a hormonal influence on baseline circulating endostatin amounts. Exercise-induced increase in endostatin is also observable in patients suffering from T2DM. Concerning vascularisation, lower endostatin levels in T2DM might be advantageous. Concerning plaque stability, lower levels might be prejudicial.

**Trial registration:**

Clinical Trial Registration-URL: http://clinicaltrials.gov/ct2/results?term=NCT01165515

## Introduction

Diabetes mellitus (T2DM) is one of the most important risk factors for cardiovascular diseases and strokes in industrialized as well as emergent countries. Men suffering from T2DM have about a 2-fold to 3-fold increased risk of developing coronary heart disease, whereas diabetic women develop a 3-fold to 7-fold risk for cardiovascular events [1]. As Mascarenhas-Melo et al. showed T2DM abrogates the protective effect of gender on non-diabetic women [2]. Due to the “AHA Classification of CVD Risk in Women”, the presence of T2DM, as a single risk factor, is sufficient to classify a woman as a “high risk of CVD”-patient [3]. T2DM induces impaired production and bioavailability of nitric oxygen (NO) leading to endothelial dysfunction promoting inflammation and subsequent atherosclerosis.

Endostatin, a heparane sulphate proteoglycan [4], comprising non-collagenous and collagenous domains [5,6], is a component of nearly all epithelial and endothelial basement membranes in the human body. It turned out to be a strong angiogenesis modulator with angiostatic effects by inhibiting proliferation [7], (VEGF-induced) migration [8] and adhesion [9] of endothelial cells and tube formation [10] leading to reduced angiogenesis.

Studies have shown that physical exercise counteracts the process of atherosclerosis in respect to its main manifestations [11-14]. Physical exercise, on a regular basis, promotes angiogenesis in the skeletal muscle due to the increased physical requirements [15]. Gu. et al., were the first to show an exercise-induced increase of endostatin in healthy subjects [16]. These findings were confirmed by Rullman et al. [17] and Suhr et al. [18]. In a further study by Gu et al., where 7 healthy male subjects who performed treadmill exercise for 4–10 minutes, showed plasma endostatin levels increased significantly. Over 30 minutes (43%), over 2 hours (73%) and 6 hours (33%) compared to baseline levels [19]. Exercise-induced increase of serum endostatin levels were also observed by Suhr et al. in short- and long-track elite runners [20]. Further to these studies, Brixius et al. observed a decrease in plasma endostatin levels in obese men following a 6-month training period. However, in all these studies only small populations of male subjects were investigated, so there remains a significant lack of data for endostatin in females.

Therefore, the primary aim of the current study was to investigate the effect of graded bicycle exercise on endostatin levels in middle-aged patients suffering from diabetes mellitus compared to a sex-and age-matched control group under a sex-specific perspective.

## Material and methods

### Included subjects

All 87 subjects were recruited at the Medical University of Vienna, had to be between 35–80 years of age, never-smokers, sparsely to moderately active (<150 min. of moderate physical exercise per week) and able to perform a bicycle stress test. Exclusion criteria for both groups were the presence of current infectious disease, anamnestic CAD (coronary artery disease) or COPD (chronic obstructive pulmonary disease). Within the control group (21 females, 24 males) only subjects not taking medication were recruited. Individuals of the T2DM-group were recruited at the outpatient clinic of the Medical University of Vienna and consisted of 22 female and 20 male patients suffering T2DM for at least 5 years with a HbA1_c_ >7% (in the course of the last check). The study was approved by the Ethical Committee of the Medical University of Vienna and all subjects provided written informed consent prior to participation.

### Bicycle stress testing

All ergometry tests were done in the early afternoon. After physical examination and a 30-minute period of rest, a needle was inserted in an anticubital vein and all included subjects had to undergo a 12-lead-ECG-controlled graded bicycle exercise test according to the ergometry protocol of the Austrian Society of Cardiology which is consistent with the protocol of the The German Society of Cardiology and the Bruce Protocol [21]. The participants were asked to reach their individual maximum workload (calculated by age, sex, height and weight) until exhaustion occured, where the workload increased every 2 minutes by 25 watts. Subjects were under permanent ECG-monitoring and blood pressure was taken at rest and every 2 minutes.

### Laboratory analysis

Subjects had to rest for 20 minutes in a quiet, peaceful room before the first blood samples were taken, to prevent any physical effort influencing endostatin baseline levels. Blood samples were taken through a venous winged infusion set prior to bicycle stress testing (baseline levels) and directly after reaching the maximum workload (stress levels). Blood samples were collected in 8 ml Z serum sep Clot-activator tubes with separating gel for the determination of endostatin. Samples were analysed with Quantikine® Human Endostatin Sandwich Enzyme Immunoassay (R&D System Inc. Minneapolis, USA).

### Statistical analysis

Statistical analysis was performed using the statistical software SPSS 20.0. Continuous and normally distributed data was described by means ± standard deviation (SD) and group differences were tested by independent sample t-test. The significance of the difference between baseline and stress endostatin was tested by paired sample t-test. All tests were performed two-sided and p ≤ 0.05 was considered significant.

## Results

Basic demographic data including the number of subjects, mean age (years), mean BMI (kg/m^2^), mean heart rate (bpm) and mean basic systolic and diastolic blood pressure (mmHg) are shown in Table 1. Although T2DM-patients were slightly older and had a higher BMI compared to the controls the differences were not significant. The same holds true for heart rate and blood pressure.

**Table 1:** Anthropometric data, heart rate and blood pressure

***Table*** 1	***Female T2DM (n = 22)***	***Female control (n = 21)***	***p-value***	***Male T2DM (n = 20)***	***Male control (n = 24)***	***p-value***
**Age (years)**	59.45 ± 9.12	58.62 ± 6.48	0.732	57.21 ± 7.80	52.80 ± 6.69	0.053
**BMI (kg/m**^**2**^**)**	27.51 ± 4.79	23.85 ± 3.58	0.054	30.12 ± 6.37	27.05 ± 2.69	0.051
**Heart rate (bpm)**	77.42 ± 11.69	74.44 ± 10.83	0.442	77.70 ± 11.70	69.75 ± 10.97	0.033
**Systoly (mmHg)**	132.00 ± 8.73	127.81 ± 10.80	0.213	132.20 ± 18.00	127.20 ± 10.24	0.287
**Diastoly (mmHg)**	82.89 ± 9.02	80.96 ± 8.36	0.462	81.60 ± 10.81	75.95 ± 6.56	0.053

Table 1: Anthropometric data, heart rate and blood pressure: Age, BMI, heart rate, systolic and diastolic blood pressure of T2DM-patients and controls. P-values show the statistical sex-specific differences between female T2DM patients and controls and male T2DM patients and controls resp. Data is presented as mean ± SD.

Routine laboratory parameters are shown in Table 2. As expected, both female and male T2DM-patients had higher glucose levels compared to controls. T2DM-patients showed lower total cholesterol, LDL-cholesterol and triglyceride levels but also lower HDL-cholesterol levels.

**Table 2:** Routine laboratory parameters

***Table*** 2	***Female T2DM (n = 22)***	***Female control (n = 21)***	***p-value***	***Male T2DM (n = 20)***	***Male control (n = 24)***	***p-value***
**Na**^**+**^	139.24 ± 2.88	139.56 ± 2.00	0.703	138.70 ± 2.79	139.15 ± 1.39	0.513
**K**^**+**^	4.59 ± 0.47	4.23 ± 0.13	0.531	4.60 ± 0.31	4.15 ± 0.27	<0.001
**Glucose (mg/dl)**	141.82 ± 43.77	93.44 ± 15.85	<0.001	140.27 ± 17.88	93.41 ± 7.46	<0.001
**Creatinin (mg/dl)**	0.96 ± 0.17	0.87 ± 0.10	0.083	0.96 ± 0.15	0.96 ± 0.97	0.912
**Erythrocytes (T/l)**	4.63 ± 0.41	4.46 ± 0.21	0.127	4.82 ± 0.36	4.83 ± 0.38	0.013
**Thrombocytes (G/l)**	247.84 ± 83.36	244.75 ± 39.99	0.893	219.83 ± 34.35	221.55 ± 45.49	0.897
**Leucocytes (G/l)**	7.21 ± 1.99	6.31 ± 1.26	0.130	6.75 ± 1.26	5.95 ± 1.60	0.099
**Haemoglobin (g/dl)**	13.17 ± 1.31	12.96 ± 0.88	0.577	14.36 ± 1.07	14.56 ± 0.93	0.474
**Hematocrit (%)**	39.41 ± 3.47	38.22 ± 2.35	0.251	41.63 ± 2.99	42.86 ± 2,50	0.179
**Total cholesterol (mg/dl)**	197.95 ± 33.47	224.69 ± 45.13	0.053	186.26 ± 43.56	230.45 ± 24.43	0.001
**HDL-cholesterol (md/dl)**	54.78 ± 9.49	72.50 ± 17.61	0.002	43.61 ± 5.95	54.90 ± 8.28	<0.001
**LDL-cholesterol (mg/dl)**	104.92 ± 26.73	125.94 ± 38.96	0.073	107.56 ± 37.92	143.41 ± 19.28	0.001
**Triglycerides (mg/dl)**	171.56 ± 72.70	131.25 ± 46.90	0.065	163.92 ± 55.32	174.69 ± 74.12	0.612

Table 2: Routine laboratory parameters of T2DM-patients and controls. P-values show the statistical sex-specific differences between female T2DM patients and controls and male T2DM patients and controls resp. Data is presented as mean ± SD.

Endostatin levels, at baseline and at maximum workload, as well as the extent to which endostatin increases immediately after the bicycle stress tests are shown in Table 3. At baseline the highest levels were shown by female control subjects and T2DM-patients (167.70 ± 18.19 vs. 145.50 ± 33.14 ng/ml; p = 0.022) followed by male controls and T2DM-patients (118.56 ± 16.41 vs. 136.40 ± 15.16 ng/ml; p = 0.031). Female T2DM-patients showed significantly higher levels of endostatin compared to male T2DM-patients (p < 0.001) and the same was observable for the control group (p < 0.001).

**Table 3:** Endostatin at baseline and after bicycle stress testing

***Table*** 3	***Female T2DM (n = 22)***	***Female control (n = 21)***	***p-value***	***Male T2DM (n = 20)***	***Male control (n = 24)***	***p-value***
**Baseline endostatin (ng/ml)**	145.50 ± 33.14	167.70 ± 18.19	0.022	106.77 ± 16.86	118.56 ± 16.41	0.031
**Stress endostatin (ng/ml)**	162.26 ± 37.25	185.46 ± 17.96	0.021	119.44 ± 15.16	136.40 ± 15.16	0.001
**Increase in endostatin -**						
**ng/ml**	16.76	17.76		12.67	17.84	
**%**	11.52	10.60		11.87	15.05	
**p-value**	<0.001	<0.001		<0.001	0.001	

Table 3: Endostatin levels at baseline and right after reaching maximum workload (stress) of T2DM-patients and controls. P-values show the statistical sex-specific differences between female T2DM patients and controls and male T2DM patients and controls resp. Data is presented as mean ± SD.

Standardized physical performance (%) during bicycle stress testing was calculated on a basis of age, sex, height and weight. Female T2DM-patients as well as male patients reached much lower performance levels compared to female and male controls (59.55 ± 11.06%/68.86 ± 13.31% vs. 105.95 ± 22.28%/97.89 ± 7.14%). Nevertheless, bicycle stress testing was associated with a significant increase in endostatin levels in both groups and sexes (p < 0.001-0.001). The highest increase was found in male controls (15.05%) followed by male T2DM-patients (11.87%), female T2DM-patients (11.52%) and female controls (10.59%). Although there were significant differences in performance, observable Pearson Correlation analysis showed no correlation between performance and basic (p = 0.110-0.841) or stress endostatin levels (p = 0.058-0.693).

## Discussion

It is well known that angiogenic factors such as VEGF play an important role in patients suffering atherosclerosis and diabetes [22] and might even be used as therapeutic targets [23]. Endostatin is suggested to be a modulator of angiogenesis with angiostatic effects and therefore a target in therapies for cancer and cardiovascular disease. The aim of the present study was to investigate baseline levels and the influence of physical exercise on serum endostatin levels in patients suffering from T2DM compared to controls. We could show for the first time that endostatin baseline levels, measured in serum, are decreased in patients suffering from T2DM compared to age- and sex-matched healthy controls. With regard to the findings by Boodhwani et al., [24] who showed that T2DM results in a profound impairment in the myocardial angiogenic response to chronic ischemia, these observations are of distinct interest. Additionally, we stated a significant exercise-induced increase in endostatin levels in T2DM patients as well as in healthy controls.

Our first study aim was to investigate the influence of acute exercise in T2DM-patients and controls. Bruserud et al. observed higher serum baseline levels of endostatin in 68–88 year old female and male people than in 18 year old male athletes and an increase in endostatin levels after physical exercise [25]. An exercise-induced increase in endostatin levels was also observed by Suhr and co-workers, who measured endostatin levels in twelve male cyclists ages 27.8 ± 5.4 years [18] and in short- and long-track elite runners [20]. Similar results were delivered by Gu et al. who measured plasma endostatin levels in seven healthy male subjects aged 18–49 years. Gu et al. [19] could show in a rat model (Sprague–Dawley rats), that continuous exercise leads to an decrease in endostatin levels (measured in tissue of skeletal muscle). Brixius et al. stated a decrease of endostatin plasma levels in 50–60 year old male overweight and untrained men after 6 months of moderate exercise 3 times/week [26]. However, Makey et al. [27] did not find an exercise-induced increase of endostatin in overweight/obese women, but in this study the participants “only” walked on a treadmill at moderate intensity. Nevertheless, considering the results of the mentioned studies both unique and regular physical exercise seems to have an impact on endostatin levels in healthy individuals, where endostatin seems to increase during exercise and to decrease after regular sportive activity. Our results support the theory of exercise-induced endostatin increase which was observable in controls as well as in patients suffering from T2DM. Although exercise-induced stress led to a significant rise in endostatin in both groups it should be mentioned that male controls showed the highest surge by 15% whereas female controls and T2DM-patients only increased by about 10–12%. Furthermore, the increase in endostatin did not seem to be connected to the extent of physical workload: Our subjects were asked to proceed with the bicycle stress test till exhaustion. Although T2DM-patients showed significantly lower performance compared to controls, there was no correlation observable between the extent of endostatin-increase, stress levels of endostatin and performance (Pearson Correlation).

Our second aim was to investigate baseline endostatin levels of T2DM-patients compared to controls with regard to sex-specific differences. Boodhwani et al. [28] measured 3,6-fold higher endostatin levels (myocardium) in Yucatan miniswine compared to controls. Sodha et al. [29] was able to show that endostatin levels are elevated 2,02-fold in diabetic patients with CAD in myocardial tissue and that their levels showed a positive correlation to blood glucose levels. Interestingly, we found significantly lower levels in both female and male patients suffering T2DM compare to controls. Additionally, female controls showed significantly higher levels compared to male controls suggesting a hormonal influence on endostatin levels. These two findings are of distinct interest.

On the one hand atherosclerotic vessels often present intra-plaque angiogenesis [30], supporting plaque expansion and leading to plaque rupture by enhancing its vulnerability [31,32]. Due to its angiostatic potential, endostatin might enable plaque stabilization by inhibiting sprouting and ingrowth of new vessels into the plaque reducing plaque neovascularisation [33,34] and therefore could reduce CVD progression. Furthermore, Wenzel et al. recently were able to show, in vitro, that endostatin reduces the vascular tonus by increasing the production of NO by endothelial cells [35]. Following this chain of thought, individuals with high levels of endostatin would be protected more effectively from CVD progression and would have more NO available.

On the other hand, Gu et al. [19] showed in rats that high endostatin levels correlate with low capillary density. Similar results were obtained from Sodha et al. [29] who found elevated endostatin levels in the myocardial tissue of diabetic patients together with a strong negative correlation to coronary collateralization. As an organ with a high metabolic demand meaning a high oxidative capacity, the myocardium is dependent on a pronounced capillary network. In this case, high endostatin levels would be prejudicial because impaired collateralization is one of the most important problems in T2DM, in particular in T2DM-patients with coronary artery disease.

With regard to our results and those of the mentioned studies, it is now quite safe to assume that acute exercise leads to an increase in endostatin in healthy individuals and also T2DM-patients nearly to the same extent. Although some studies found increased amounts in animals and patients suffering from T2DM, out data do not support this thesis. To our knowledge, the present study is the first one showing an exercise-induced surge in serum endostatin also in T2DM-patients and a sex-specific difference in baseline endostatin levels with female T2DM-patients and controls showing higher levels than male individuals.

Endostatin levels seem to be dependent on a great number of factors such as sex, age, race, underlying diseases, level of physical fitness and many more. Additionally, the medium in which endostatin is analysed (serum, plasma, muscle tissue, myocardial tissue, arterial, venous…) seems to be of importance in determining its scope of action. Further studies with higher numbers of participants are needed to 1) investigate the influence of regular physical exercise on endostatin release and 2) study the role of endostatin in diabetes. At the present time it seems that endostatin is involved in various mechanisms dealing with angiogenesis and therefore it would be advisable to regard it as modifier of angiogenesis with possible influence in both physiological and pathological angiogenesis.

## Conclusion

This is the first study showing an exercise-induced increase in patients suffering T2DM. There seems to be a sex-specific difference in baseline endostatin levels: Healthy female individuals as well as female T2DM-patients showed higher circulating serum endostatin levels compared to males. However, the reason for this sex-specific difference remains unclear but might be caused by hormonal reasons.

## Competing interests

The authors declare that they have no competing interests.

## Authors’ contributions

MS acted as “clinical investigator”. He carried out the bicycle stress tests, physical examinations, taking blood samples and statistical analysis. DD was involved in planning the project in course of her diploma thesis and involved in carrying out the bicycle stress tests in the diabetes group. SK was involved in planning the project in course of her diploma thesis and involved in carrying out the bicycle stress tests in the diabetes group. MFZ carried out laboratory storing, preparation and analysis of endostatin samples. JSJ acted as “senior investigator” planning and observing the project. All authors read and approved the final manuscript.
